# Formation of Pyrazines in Maillard Model Systems: Effects of Structures of Lysine-Containing Dipeptides/Tripeptides

**DOI:** 10.3390/foods10020273

**Published:** 2021-01-29

**Authors:** Furong Wang, Hailiang Shen, Ting Liu, Xi Yang, Yali Yang, Yurong Guo

**Affiliations:** 1 College of Food Engineering and Nutritional Science, Shaanxi Normal University, Xi’an 710000, China; wangfurong@snnu.edu.cn (F.W.); tingliu@snnu.edu.cn (T.L.); yangxi@snnu.edu.cn (X.Y.); yrguo730@snnu.edu.cn (Y.G.); 2 National Research & Development Center of Apple Processing Technology, Shaanxi Normal University, Xi’an 710000, China; 3 Citrus Research Institute, Southwest University, Chongqing 400715, China; shl2021@email.swu.edu.cn; 4 Citrus Research Institute, Chinese Academy of Agricultural Science, Chongqing 400715, China

**Keywords:** peptides, FAAs, Maillard reaction models, volatile formation, pyrazines

## Abstract

At present, most investigations involving the Maillard reaction models have focused on free amino acids (FAAs), whereas the effects of peptides on volatile products are poorly understood. In our study, the formation mechanism of pyrazines, which were detected as characteristic volatiles in sunflower seed oil, from the reaction system of glucose and lysine-containing dipeptides and tripeptides was studied. The effect of the amino acid sequences of the dipeptides and tripeptides on pyrazine formation was further highlighted. Four different dipeptides and six tripeptides were selected. The results showed that the production of pyrazines in the lysine-containing dipeptide models was higher than that in the tripeptide and control models. Compounds 2,5(6)-Dimethylpyrazine and 2,3,5-trimethylpyrazine were the main pyrazine compounds in the dipeptide models. Furthermore, the *C*- or *N*-terminal amino acids of lysine-containing dipeptides can exert an important effect on the formation of pyrazines. In dipeptide models with lysine at the *C*-terminus, the content of total pyrazines followed the order of Arg−Lys > His−Lys; the order of the total pyrazine content was Lys−His > Lys−Arg in dipeptide models with *N*-terminal lysine. Additionally, for the tripeptide models with different amino acid sequences, more pyrazines and a greater variety of pyrazines were detected in the tripeptide models with *N*-terminal lysine/arginine than in the tripeptide models with *N*-terminal histidine. However, the total pyrazine content and the percentage of pyrazines in the total volatiles were similar in the tripeptide models with the same amino acids at the *N*-terminus. This study clearly illustrates the ability of dipeptides and tripeptides containing lysine, arginine and histidine to form pyrazines, improving volatile formation during sunflower seed oil processing.

## 1. Introduction

The Maillard reaction is a complex chemical reaction between a carbonyl compound and an amino group (e.g., amine, amino acid, peptide, or protein) under heating conditions [1]. It contributes largely to the formation of aroma compounds in food products [2], which further affects the taste, color, aroma, and nutrition of foods. In the past few decades, researchers have established numerous Maillard model systems to understand the reaction process and products. In these models, free amino acids (FAAs) and carbonyl compounds are the most studied candidates [1,3,4,5,6]. Given that peptides and/or proteins are much more abundant in foods than FAAs [7,8,9], it seems to be of great significance to explore Maillard model systems consisting of peptides and carbonyl compounds [10,11,12]. Compared with the FAA-containing Maillard models, the specific volatile products from the peptide-containing Maillard models were higher [13]. For instance, pyrrolizines were detected in the proline-containing dipeptide model and considered proline-specific volatile compounds [14]. The 2(lH)-pyrazinones were only detected in the Gly-containing peptide Maillard models but were not formed in FAA models [15]. Because the structure of glycine is relatively simple, glycine-containing peptides are commonly used as the main model peptides [16]. However, peptides composed of other amino acids can generate various specific volatile compounds [17,18,19,20,21,22,23].

As typical Maillard reaction products (MRPs), pyrazines are the major aromatic contributor to baked, roasted, meaty and popcorn-like foods [24,25]. It is also a typical flavor compound in sunflower seed oil [25]. Some references reported that pyrazines were detected in high amounts in the Maillard reaction model containing lysine, especially lysine-containing peptide models [3,26,27,28,29,30]. Van Lancker et al. investigated the effect of different dipeptides with lysine at the *N*-terminus (Lys−X) or the *C*-terminus (X−Lys) on volatile formation (X = Pro, Leu, Ala, Val, Ser and Gly), and the different neighboring amino acids had significant effects on volatile formation, especially pyrazines [29,30]. Additionally, it is well known that lysine, arginine and histidine are very important essential amino acids. According to our previous research, the contents of lysine, arginine and histidine decreased significantly during the baking process of sunflower seeds, and they played an important role in the formation of typical flavor compounds of sunflower seed oil during processing. Furthermore, it was reported that in the Maillard reaction, polypeptides could be degraded into dipeptides and tripeptides and further form volatiles [31]. However, there are few studies on the influence of dipeptides and tripeptides containing lysine, arginine and histidine on flavor formation. Thus, in our study, we selected dipeptides and tripeptides containing lysine, arginine and histidine as the precursors of the Maillard reaction to analyze typical flavor (pyrazine) formation in sunflower seed oil.

In the present work, to understand the pyrazine formation mechanism in sunflower seed oil, we reacted dipeptides containing lysine at the *C*-terminus (X−Lys) or the *N*-terminus (Lys−X) and tripeptides with different structures with glucose (X = Arg or His) and monitored the formation of pyrazines using the corresponding FAAs (free amino acids) as the control. The possible formation mechanism of pyrazines between peptides and glucose is also discussed. The dipeptide and tripeptide Maillard reaction models clearly illustrate the contribution of peptides to the formation of flavor compounds, especially pyrazines, improving the knowledge of flavor formation in sunflower oil processing to obtain higher-quality products.

## 2. Materials and Methods

### 2.1. Chemicals

L-lysine (≥98%), L-histidine (≥98%), and L-arginine (≥98%) were purchased from Shanghai Yuanye Bio-Technology Co., Ltd., China. Lys−Arg (≥98%), Lys−His (≥98%), Arg−Lys (≥98%), His−Lys (≥98%), Lys−Arg−His (≥98%), Lys−His−Arg (≥98%), His−Lys−Arg (≥98%), His−Arg−Lys (≥98%), Arg−His−Lys (≥98%) and Arg−Lys−His (≥98%) were purchased from Asiapeptide (Wuxi, China). Glucose and methanol were purchased from Tianjin Kemiou Chemical Reagent Co. Ltd. (Tianjin, China). NaOH was purchased from Tianjin Tianli Chemical Reagent Co. Ltd. (Tianjin, China). Tridecane was purchased from Shanghai Yuanye Bio-Technology Co. Ltd. (Shanghai, China).

### 2.2. Preparation of Maillard Model Reaction Systems

Equal masses of dipeptides (100 mg) and glucose (100 mg) were mixed to prepare a dipeptide + glucose mixture. Likewise, the individual FAAs (50 mg for each amino acid) of the dipeptides were mixed with glucose (100 mg) to serve as a control. Similarly, tripeptide systems were prepared according to the same protocol, where 33.3 mg of individual FAAs of the tripeptides were mixed with 100 mg of glucose to act as a control. The above mixtures were dissolved in 10 mL of distilled water. The pH was adjusted to 8.0 with NaOH (6 N). The mixtures were transferred to 20 mL solid-phase microextraction (SPME) vials (Nanjing Daobang Biotechnology Co., Ltd., Nanjing, China) and heated in a stirred oil bath at 140 °C for 90 min, after which the vials were immediately cooled in a 4 °C water bath. The samples were adjusted to pH 7.0 for SPME analysis. Twenty microliters of tridecane solution in methanol (10 µg/mL) was added to each sample as an internal standard.

### 2.3. Determination of Volatile Compounds (Headspace-SPME–GC–MS)

The samples were equilibrated at 45 °C for 20 min in a water bath before headspace-SPME analysis. The SPME fiber was a DVD (Divinylbenzene) /Car/PDMS (Polydimethylsiloxa) fiber (50/30 µm thickness, Supelco, Bornem). Then, the volatile compounds were extracted at 50 °C for 30 min. GC–MS analyses were performed by using an Agilent 8890 GC coupled with an Agilent 5977B quadrupole mass selective detector (MSD, Agilent Technologies, Diegem, Belgium) with a Varian DB-1701 capillary column (30 m length × 0.25 mm i.d.; 0.25 µm film thickness). The working conditions of GC–MS were as follows: the transfer line to MSD (Mass Selective Detector) was maintained at 250 °C; the carrier gas (He) flow rate was 1.0 mL/min; the electron ionization (EI) was 70 eV; the scanned acquisition parameter ranged from 30 to 550 m/z; the initial oven temperature was 40 °C for 2 min; the temperature program was increased from 40 to 100 °C at 10 °C/min and held for 5 min and then raised to 220 °C at a rate of 10 °C/min and held for 15 min, and the equilibrium time was 0.5 min. The injection port was in split mode, and the split ratio was 30:1.

### 2.4. Identification and Quantification of Volatile Compounds

The identification of volatile compounds was based on the comparison of mass spectra of the volatile compounds and the mass spectrum with mass spectral libraries (NIST 2017). The compounds were quantified by peak area normalization. The reference formula is as follows:

$$C=\frac{S}{S_{i}}\times C_{i}/m$$

where C represents the concentration of the unknown compounds (µg/g); *C*_i_ represents the content of the internal standard (µg); S represents the GC–MS peak area (×10^6^) of the unknown compounds; S_i_ represents the peak area (×10^6^) of the internal standard, and m represents the sample mass (g).

### 2.5. Statistical Analysis

All experiments were carried out three times, and the data were analyzed by SPSS Statistics Version 22 software (IBM, New York, NY, USA). Principal component analysis (PCA) was performed to determine the relationships among variables. Correlations were analyzed by using STAT-ITCF statistical software (ITCF, Bordeaux, France).

## 3. Results

### 3.1. Pyrazine Formation in the Maillard Reaction Model Containing Arg/His−Lys Dipeptides and Glucose

According to the results of our preliminary experiments, a heating protocol of 140 °C for 90 min was selected as providing suitable conditions for the reaction of Maillard model systems. The peptides were mixed with glucose and reacted at pH 8.0 and 140 °C for 90 min. The volatile compounds were analyzed in Maillard reaction models containing Arg/His−Lys dipeptides and glucose using their corresponding FAAs as controls, which were designated Control _(Arg + Lys)_ and Control _(His + Lys)_, respectively. Note that we did not use phosphoric buffer to adjust the pH of the reaction system because the anion species of the buffer can catalyze phosphate ions, further affecting the reaction [32]. In addition, considering that weak alkaline conditions can facilitate the formation of pyrazine, the pH values of all Maillard systems were adjusted to 8.0 before the start of the reaction [33]. However, the pH decreased rapidly (pH 4–5) because of acid formation during the reaction period [34].

Table 1 shows the formation of pyrazines in the different dipeptides with lysine at the *C*-terminus (X−Lys), corresponding FAAs and glucose models. According to the GC–MS analysis results, 20 and 10 pyrazines were detected in the Arg−Lys and His−Lys models, respectively, while 14 and 12 pyrazines were detected in Control _(Arg + Lys)_ and Control _(His + Lys)_, respectively. The proportion of pyrazines in the Arg−Lys and His−Lys model systems in the total GC–MS peak area was higher than that in the control. This result indicated that the specificity of volatiles from the lysine-dipeptide models was higher than that from the FAA models.

In our study, 2,5-dimethylpyrazine and 2,6-dimethylpyrazine were always eluted together, and their elution peaks were not separated satisfactorily. Thus, 2,5-dimethylpyrazine and 2,6-dimethylpyrazine were defined as 2,5(6)-dimethylpyrazine [28]. Compared with the flavor compounds in Control _(Arg + Lys)_ and Control _(His + Lys)_, more pyrazines were generated in the dipeptide models, especially 2,5(6)-dimethylpyrazine and 2,3,5-trimethylpyrazine. This was probably due to the catalysis of the Amadori rearrangement in the dipeptide/sugar adduct [35]. However, pyrazine and 2-methylpyrazine were detected as the main pyrazine compounds in the Control _(His + Lys)_ and Control _(Arg + Lys)_ models, which agreed with the statement of Negroni et al. [36]. Hwang et al. [26] reported that the *ε*-amino group of lysine that should produce pyrazine and methylpyrazine in the presence of *FFAs* becomes less reactive when combined with another amino acid to form peptides. This may explain the generation of more pyrazine and methylpyrazine in the control models. Similar results were also reported by Van Lancker et al. [30]. Moreover, 2-ethylpyrazine was only detected in the Arg−Lys and His−Lys models, and the content was low (0.32 µg/g and 0.05 µg/g, respectively). The compound 3-Ethyl-2,5-dimethylpyrazine, an amino acid-specific pyrazine, was detected to have a high content in the Control _(Arg + Lys)_ and Control _(His + Lys)_ models (2.5 µg/g and 0.8 µg/g, respectively), and it was also formed in the corresponding dipeptide-containing systems, but the content was low (0.93 µg/g and 0.08 µg/g).

The total pyrazine yield detected was much more evident in the Arg−Lys and glucose model (13.12 µg/g) than in the His−Lys model (5.54 µg/g). Ethenylpyrazine, 2-methyl-6-propylpyrazine and 2-ethenyl-6-methylpyrazine were only produced in the Arg−Lys or Control _(Arg + Lys)_ model and were not detected in the His−Lys or Control _(His + Lys)_ model. This result suggested that different *N*-terminal amino acids of dipeptides could exert an important effect on the formation of pyrazines, which may be due to the different reactivity of the *N*-terminal amino acid side chain or the degree of hydrolysis of the peptide bond [30].

### 3.2. Pyrazine Formation in the Maillard Reaction Model of Lys−Arg/His Dipeptides and Glucose

The *C*-terminal amino acids of dipeptides were varied to further study the effect of the structures of Lys-containing dipeptides on pyrazine formation. As shown in Table 2, only nine pyrazines were detected in the Lys−Arg model, and 2,5(6)-dimethylpyrazine and 2,3,5-trimethylpyrazine had the highest contents, while the Lys−His model generated 18 pyrazines, and it produced more pyrazines than that in the Lys−Arg model. Only a small amount of ethenylpyrazine was detected in the Lys−Arg and Control _(Arg + Lys)_ models compared with the pyrazine compounds in the Lys−His and Control _(His + Lys)_ models.

In contrast to the Arg−Lys model, the total content of pyrazines was lower in the Lys−Arg model (7.04 µg/g), and the percentage of pyrazines in the total GC–MS peak area was also lower (23.76%). The percentage of pyrazines in the total volatiles of the Lys−Arg model was higher than that of the Control _(Arg + Lys)_ model_._ These results indicated that the percentage of pyrazines in the total volatile products followed the order of Arg−Lys (73.83%) > Lys−Arg (23.76%) > Control _(Arg + Lys)_ (22.10%). Additionally, the total pyrazines of the Lys−His model were higher than those in the His−Lys and Control _(His + Lys)_ models, and the percentage of pyrazines in the total volatiles followed the order of Lys−His (84.10%) > His−Lys (56.10%) > Control _(His + Lys)_ (20.71%). The Lys−Arg and Lys−His Maillard models had higher contents of 2,5(6)-dimethylpyrazine and 2,3,5-trimethylpyrazine in comparison with their corresponding FAA Maillard models (Control _(Arg + Lys)_ and Control _(His + Lys)_), whereas pyrazine, 2-methylpyrazine and 3-ethyl-2,5-dimethylpyrazine were detected more in the Control _(Arg + Lys)_ and Control _(His + Lys)_ models, which was similar to the results described in Table 1.

### 3.3. Pyrazine Formation in the Maillard Reaction Models of Glucose and Tripeptides with Different Structures

The Maillard reaction models of glucose and lysine-containing tripeptides with different structures (i.e., Lys-Arg-His, Lys-His-Arg, Arg-His-Lys, Arg-Lys-His, His-Lys-Arg and His-Arg-Lys) were also studied, and the results are listed in Table 3. Their corresponding FAAs were used as a control (Control _(Lys + Arg + His)_). Compared to the total pyrazine content in the dipeptide Maillard models, the total pyrazine content in the tested tripeptide models was lower. However, the proportion of pyrazines in the total volatiles was large in the most tripeptide models, indicating that the few volatiles were generated by the tripeptide models and the specificity of the volatiles was relatively high. The contents of pyrazine and 2-methylpyrazine were higher in Control _(Lys +Arg + His)_ (0.12 µg/g and 1.90 µg/g), which was similar to the results of the dipeptide models. The compound 3-Ethyl-2,5-dimethylpyrazine was detected at a higher content in the Control _(Lys +Arg + His)_ model than in the tripeptide models, which was consistent with the trends shown in Table 1 and Table 2. However, 2,5(6)-dimethylpyrazine and 2,3,5-trimethylpyrazine were produced more in the Control _(Lys +Arg + His)_ model (4.68 µg/g and 0.77 µg/g) than in all tested tripeptide models. This result was different from the trend of the tested dipeptide Maillard models, which could be due to the degree of hydrolysis of peptide bonds. Furthermore, 2-ethylpyrazine and 2-ethyl-3,5-dimethylpyrazine were only detected in the tripeptide models, while ethenylpyrazine was only produced in the Control _(Lys +Arg + His)_ model.

As shown in Table 3, the total pyrazine contents of the tripeptide models with lysine at the *N*-terminus (Lys-Arg-His and Lys-His-Arg) were similar (6.86 µg/g and 4.20 µg/g). Likewise, the total pyrazine contents of the tripeptide models with histidine at the *N*-terminus (His-Lys-Arg and His-Arg-Lys) (1.77 µg/g and 1.70 µg/g) and arginine at the *N*-terminus (Arg-His-Lys and Arg-Lys-His) (4.89 µg/g and 5.01 µg/g) were also close. In addition, the percentage of pyrazines in the total volatiles was also similar in the tested tripeptide models with the same amino acid at the *N*-terminus. These results suggested that the difference in *C*-terminal amino acids had no significant effect on pyrazine formation (*p <* 0.05). However, a significant difference was found in the effect of tripeptide models with different *N*-terminal amino acids on pyrazine formation (*p <* 0.05). For instance, the contents of total pyrazines in the His-Arg-Lys and His-Lys-Arg models were the lowest among all tested tripeptide Maillard models. Notably, compared with the Lys-Arg-His and Lys-His-Arg models, the Arg-His-Lys and Arg-Lys-His models had higher contents of 2,5(6)-dimethylpyrazine, but the content of 2,3,5-trimethylpyrazine was lower, which was not consistent with the pyrazine formation of dipeptide Maillard models. However, no relevant mechanistic study is available to explain this phenomenon, which may be due to the different composition of amino acid residues of peptides or the degree of hydrolysis of peptide bonds [31].

### 3.4. Principal Component Analysis

**Figure 1:**
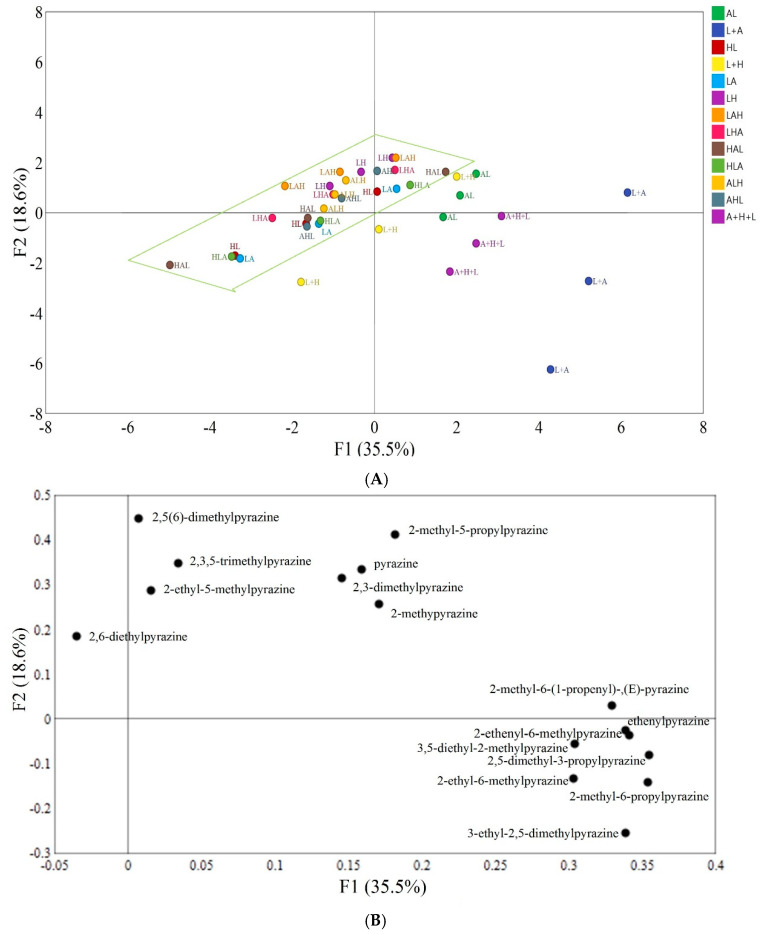
(**A**). The results of principal component analysis for different reaction models. AL: Arg-Lys; L + A: Control _(Arg + Lys)_; HL: His-Lys; L + H: Control _(His + Lys)_; LA: Lys-Arg; LH: Lys-His; LAH: Lys-Arg-His; LHA: Lys-His-Arg; HAL: His-Arg-Lys; HLA: His-Lys-Arg; ALH: Arg-Lys-His; AHL: Arg-His-Lys; A + H + L: Control _(Lys + Arg + His)._ (**B**). Principal component analysis (PCA) of the pyrazines produced from different reaction models.

Principal component analysis (PCA) was performed on the data collected by HS–SPME–GC/MS. In Figure 1A, the distance between points reflected the difference in data between different reaction model groups. The variance contribution rates of principal component 1 (PC1) and principal component 2 (PC2) were 35.5 and 18.6%, respectively, indicating that PCA could reflect most of the sample information and distinguish the results of the tested reaction models. As shown in Figure 1A, L + H (Control _(His + Lys)_) and L+A (Control _(Arg + Lys)_) were mainly distributed in the negative half axis of PC2, while the dipeptide models were mainly distributed in the positive axis of PC2. This indicated that there was a significant difference between the dipeptide models and their corresponding FAA models. Similarly, A + H + L (Control _(Lys +Arg + His)_) was mainly distributed in the fourth quadrant, while the other tripeptide models were mainly distributed in the second quadrant and the third quadrant, indicating that there was also a significant difference between the tripeptide models and their corresponding FAA models. Thus, our results proved that lysine-containing peptides and FAAs have different effects on the formation of pyrazines in the Maillard reaction and that dipeptides and tripeptides play an important role in pyrazine generation. However, the distribution concentration of dipeptide and tripeptide models was shown in Figure 1A, which indicated that the influence of the tested dipeptide and tripeptide Maillard models on pyrazine formation was not significant (*p* *<* 0.05).

Figure 1B shows the distribution of volatile components in the two principal components. Compounds 2,5(6)-Dimethylpyrazine, 2,3,5-trimethylpyrazine, pyrazine, 2-methylpyrazine, 2-ethyl-5-methylpyrazine, 2,3-dimethylpyrazine and 2-methyl-5-propylpyrazine, 3-ethyl-2,5-dimethylpyrazine, 2-methyl-6-propylpyrazine, 2-ethyl-6-methylpyrazine, 2,5-diethyl-3-propylpyrazine, 3,5-diethyl-2-methylpyrazine, ethenylpyrazine, 2-ethenyl-6-methylpyrazine, and 2-methyl-6-(1-propenyl)-,(E)-pyrazine had positive scores for PC1, and most of these pyrazines showed higher amounts in the Arg-Lys, Control _(Arg + Lys)_ and Control _(Lys + Arg + His)_ models. In terms of the distribution of these pyrazines along PC2, 2,6-diethylpyrazine, 2,5(6)-dimethylpyrazine, 2,3,5-trimethylpyrazine, pyrazine, 2-methylpyrazine, 2-ethyl-5-methylpyrazine, 2-methyl-5-propylpyrazine, 2-methyl-6-(1-propenyl)-,(E)-pyrazine and 2,3-dimethylpyrazine were distributed on the positive side of PC2, indicating their higher content in the Lys-Arg-His, Arg-Lys-His and Lys-His models. Regarding the first and second PCs, 3-ethyl-2,5-dimethylpyrazine, 2-methyl-6-propylpyrazine, 2-ethyl-6-methylpyrazine, 2,5-diethyl-3-propylpyrazine, 3,5-diethyl-2-methylpyrazine, ethenylpyrazine and 2-ethenyl-6-methylpyrazine were distributed along the negative side of PC2 and the positive side of PC1, indicating their higher contents in the Control _(Lys +Arg + His)_ models but low content in all peptide models. Furthermore, 2,6-diethylpyrazine had negative scores for PC1 and PC2, which indicated that 2,6-diethylpyrazine had a higher content in the Lys-Arg-His model and Arg-Lys-His model.

In addition, according to the PCA, pyrazines were divided into three broad categories. The compounds 2,5(6)-Dimethylpyrazine, 2,3,5-trimethylpyrazine, 2-ethyl-5-methylpyrazine, pyrazine, 2,3-dimethylpyrazine, 2-methylpyrazine and 2-methyl-5-propylpyrazine were classified into the first category. This is because they have higher contents in the Arg-Lys model than that in the His-Lys model and the dipeptide models with *N*-terminal lysine (expect 2-ethyl-5-methylpyrazine). Compound 2,6-Diethylpyrazine was classified into the second category because it has a higher content in the Lys-Arg-His and Arg-Lys-His models than that in the other tripeptide models. Moreover, pyrazines distributed in the fourth quadrant were found to show low contents in all dipeptide and tripeptide models; thus, they were classified into the third category.

## 4. Discussion

Pyrazines are the characteristic products of MRPs, which are also the typical volatile substances of edible vegetable oil, especially sunflower seed oil, and mostly produced during heat processing [37]. They are also significantly contributed to the baked, roasted, meaty and nut-like aroma of some heated foods [24,25,38,39]. Pyrazines, mainly 2,5-dimethylpyrazine, are the key volatile components of baked sunflower seeds [40].

The general mechanism of pyrazine formation based on FAAs involves decarboxylation and hydrolysis of the imine. First, α-aminoketones are generated by the Strecker degradation reaction. In this step, a dicarbonyl compound, produced by the degradation of glucose, reacts with an amino acid, leading to the formation of a Strecker aldehyde and an α-aminoketone. It is worth mentioning that decarboxylation of the intermediate product through a cyclic transition state is the key process in this step. Subsequently, two α-aminocarbonyl compounds are condensed to form the intermediate dihydropyrazine. Then, the intermediate dihydropyrazine is deprotonated to produce dihydropyrazine anions that react with a carbonyl compound to form alkylpyrazines in an aldol-type reaction [41]. The formation of the amino acid-specific pyrazine is thought to be the product generated from the reaction of the intermediate dihydropyrazine with the Strecker aldehyde of a specific amino acid. During the reaction of dihydropyrazine anions and a carbonyl compound, 3-ethyl-2,5-dimethylpyrazine is formed only when the reacting carbonyl compound is Strecker aldehyde of alanine [29].

**Figure 2:**
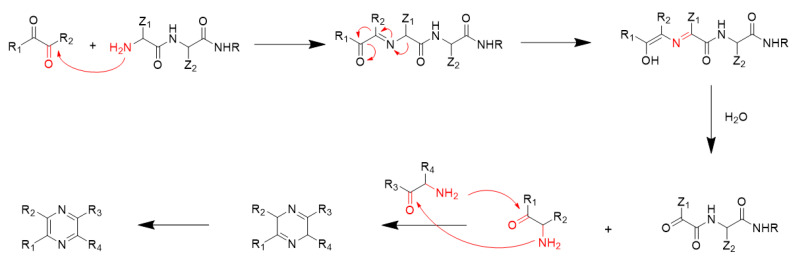
Possible formation mechanism of pyrazines by the reaction of peptides and α-dicarbonyl compounds [29].

However, typical Strecker degradation is involved in decarboxylation; peptides lack free carboxyl groups at the α-carbon, so peptides cannot undergo the typical Strecker degradation reaction to produce α-aminoketones, which further results in the formation of pyrazines. A mechanism for describing pyrazine formation in dipeptides and α-dicarbonyl compounds has been proposed by Van Lancker et al. [29] (Figure 2). First, based on the FAAs, an α-dicarbonyl compound is degraded by glucose and reacts with a dipeptide to form an α-ketoimine. Afterwards, deprotonation occurs at the α-position of the amide moiety followed by a 1,5-H-shift, leading to enolization of the carbonyl group of α-ketoimine and the formation of 4-hydroxy-2-azadiene. The imino moiety of 2-azadiene is hydrolyzed to produce an α-aminoketone. Additionally, instead of forming a Strecker aldehyde, a complex α-ketoamide is produced and further reacts with the α-aminoketone to form pyrazines. In the model of dipeptides or tripeptides, we also detected a small amount of the amino acid-specific pyrazine (3-ethyl-2,5-dimethylpyrazine). This may be because in this reaction model, dipeptides or tripeptides were slightly hydrolyzed to produce free amino acids, which further participated in the formation of 3-ethyl-2,5-dimethylpyrazine [30].

The content of pyrazines was higher in the tested dipeptide and tripeptide models than in their corresponding FAA systems. In terms of pyrazine varieties, 2,5(6)-dimethylpyrazine and 2,3,5-trimethylpyrazine were the two dominant pyrazines. This phenomenon may be due to the catalysis of the Amadori rearrangement in the dipeptide/sugar adduct, as reported by De Kok and Rosing [35]. Zou et al. [42] also reported that in the reaction of dipeptides with xylose, Amadori-type conjugates could affect the activity of C2, C3, and C4 sugar fragments and further influence the production of pyrazines. They found that the reaction of xylose with dipeptides led to the formation of an imine and peptide-Amadori compounds via Amadori rearrangement. This intermediate product results in the formation of a series of Amadori-type conjugates, which can contribute to the formation of pyrazines.

## 5. Conclusions

In summary, Maillard reaction models between glucose and peptides were established in our study, with their corresponding FAA models as controls. The formation of volatile compounds was analyzed in these Maillard models. Pyrazines were the main volatiles in the tested Maillard models. The content of pyrazines was higher in the dipeptide models than in the tripeptide and control models. The *C*- or *N*-terminal amino acids of lysine-containing dipeptides can exert an important effect on the formation of pyrazines. In dipeptide models with lysine at the *C*-terminus, the content of total pyrazines followed the order of Arg-Lys > His-Lys, whereas the order of total pyrazine content was Lys-His > Lys-Arg in dipeptide models with *N*-terminal lysine. Compounds 2,5(6)-Dimethylpyrazine and 2,3,5-trimethylpyrazine were the main pyrazine compounds in the dipeptide models. Additionally, for the tripeptide models with different amino acid sequences, more pyrazines as well as a greater variety of pyrazines were detected in the tripeptide models with *N*-terminal lysine/arginine than in the tripeptide models with *N*-terminal histidine. However, the total pyrazine content and the percentage of pyrazines were similar in the tripeptide models with the same *N*-terminal amino acids. It can be seen that *N*-terminal amino acids in the tripeptide models can exert an important effect on pyrazine formation. These results demonstrated that the structure of peptides was important for pyrazine formation during the Maillard reaction, and this should be given more attention in heat-treated foods. The awareness of pyrazine formation in sunflower seed oil processing based on Maillard reaction precursors needs to be increased to obtain higher-quality sunflower seed oil products.

## Figures and Tables

**Table 1:** Pyrazines contents (µg/g) in the model system (glucose with X−Lys dipeptides) and in the Control _(Arg + Lys)_ and Control _(His + Lys)_ (*p <* 0.05).

No.	RT (min)	Substances	Arg–Lys	Lys + Arg	His–Lys	Lys + His
1	9.556	pyrazine	0.10 ± 0.02	0.31 ± 0.045	0.02 ± 0.001	0.07 ± 0.004
2	10.907	2-methylpyrazine	1.05 ± 0.03	2.47 ± 0.44	0.14 ± 0.03	1.06 ± 0.06
3	12.649	2,5(6)-dimethylpyrazine	5.23 ± 0.99	0.36 ± 0.007	4.05 ± 0.86	1.02 ± 0.05
4	13.057	2-ethylpyrazine	0.32 ± 0.04	ND	0.05 ± 0.002	ND
5	13.516	2,3-dimethylpyrazine	0.16 ± 0.008	0.37 ± 0.004	0.01 ± 0.001	0.08 ± 0.003
6	14.858	2-ethyl-6-methylpyrazine	1.57 ± 0.06	1.14 ± 0.021	0.06 ± 0.002	0.23 ± 0.06
7	15.092	2-ethyl-5-methylpyrazine	1.35 ± 0.11	0.83 ± 0.06	0.43 ± 0.06	1.20 ± 0.04
8	15.525	2,3,5-trimethylpyrazine	1.26 ± 0.08	0.59 ± 0.08	0.68 ± 0.05	0.07 ± 0.003
9	15.975	2-(N-propyl)pyrazine	0.02 ± 0.004	ND	ND	ND
10	16.484	2,6-diethylpyrazine	0.08 ± 0.002	ND	ND	ND
11	16.534	ethenylpyrazine	0.02 ± 0.001	0.27 ± 0.02	ND	ND
12	16.834	3-ethyl-2,5-dimethylpyrazine	0.93 ± 0.05	2.50 ± 0.18	0.08 ± 0.003	0.80 ± 0.01
13	17.334	2,5-diethylpyrazine	0.09 ± 0.004	ND	0.02 ± 0.001	0.18 ± 0.02
14	17.376	2-ethyl-3,5-dimethylpyrazine	0.17 ± 0.03	ND	ND	ND
15	17.476	2-methyl-6-propylpyrazine	0.10 ± 0.002	0.10 ± 0.008	ND	ND
16	17.859	2-methyl-5-propylpyrazine	0.05 ± 0.007	ND	ND	ND
17	18.201	2-ethenyl-6-methylpyrazine	0.05 ± 0.003	0.24 ± 0.03	ND	ND
18	18.359	3,5-diethyl-2-methylpyrazine	0.30 ± 0.008	0.38 ± 0.07	ND	0.13 ± 0.03
19	18.960	2,5-dimethyl-3-propylpyrazine	0.10 ± 0.001	0.19 ± 0.02	ND	0.12 ± 0.03
20	19.685	2-methyl-6-(1-propenyl)-,(E)-pyrazine	0.07 ± 0.002	0.34 ± 0.006	ND	0.15 ± 0.01
		Total pyrazines	13.12 ± 0.96	10.09 ± 0.37	5.54 ± 0.23	5.11 ± 0.38
		Pyrazines (%of total GC-MS peak area)	73.83	22.10	56.94	20.71

ND represents no substance was detected.

**Table 2:** Pyrazines contents (µg/g) in the model system (glucose with Lys-X dipeptides) and in the Control _(Arg + Lys)_ and Control _(His + Lys)_ (*p <* 0.05).

No.	RT (min)	Substances	Lys-Arg	Lys + Arg	Lys-His	Lys + His
1	9.556	pyrazine	0.10 ± 0.004	0.31 ± 0.09	0.05 ± 0.003	0.07 ± 0.002
2	10.907	2-methylpyrazine	0.41 ± 0.07	2.47 ± 0.04	0.90 ± 0.02	1.06 ± 0.03
3	12.649	2,5(6)-dimethylpyrazine	5.03 ± 0.78	0.36 ± 0.05	4.73 ± 0.14	1.02 ± 0.04
4	13.057	2-ethylpyrazine	ND	ND	0.15 ± 0.02	ND
5	13.516	2,3-dimethylpyrazine	0.02 ± 0.003	0.37 ± 0.07	0.54 ± 0.008	0.08 ± 0.004
6	14.858	2-ethyl-6-methylpyrazine	ND	1.14 ± 0.11	0.22 ± 0.006	0.23 ± 0.01
7	15.092	2-ethyl-5-methylpyrazine	0.03 ± 0.005	0.83 ± 0.04	1.98 ± 0.03	1.20 ± 0.07
8	15.525	2,3,5-trimethylpyrazine	1.26 ± 0.02	0.59 ± 0.06	0.86 ± 0.007	0.07 ± 0.002
9	16.484	2,6-diethylpyrazine	ND	ND	0.03 ± 0.005	ND
10	16.534	ethenylpyrazine	0.04 ± 0.006	0.27 ± 0.01	ND	ND
11	16.834	3-ethyl-2,5-dimethylpyrazine	ND	2.50 ± 0.03	0.29 ± 0.02	0.80 ± 0.05
12	17.334	2,5-diethylpyrazine	ND	ND	0.23 ± 0.06	0.18 ± 0.008
13	17.376	2-ethyl-3,5-dimethylpyrazine	ND	ND	0.26 ± 0.09	ND
14	17.476	2-methyl-6-propylpyrazine	ND	0.10 ± 0.01	ND	ND
15	17.859	2-methyl-5-propylpyrazine	ND	ND	0.04 ± 0.003	ND
16	18.201	2-ethenyl-6-methylpyrazine	ND	0.24 ± 0.14	0.01 ± 0.001	ND
17	18.359	3,5-diethyl-2-methylpyrazine	ND	0.38 ± 0.09	0.21 ± 0.03	0.13 ± 0.003
18	18.401	(1-methylethenyl)pyrazine	0.09	ND	ND	ND
19	18.960	2,5-dimethyl-3-propylpyrazine	ND	0.19 ± 0.02	0.02 ± 0.005	0.12 ± 0.01
20	19.027	2,3-diethyl-5-methylpyrazine	ND	ND	0.02 ± 0.002	ND
21	19.685	2-methyl-6-(1-propenyl)-,(E)-pyrazine	0.06 ± 0.003	0.34 ± 0.09	0.09 ± 0.001	0.15 ± 0.04
		Total pyrazines	7.04 ± 0.82	10.09 ± 0.98	10.63 ± 1.22	5.11 ± 0.85
		Pyrazines (%of total GC–MS peak area)	23.76	22.10	84.10	20.71

ND represents no substance was detected.

**Table 3:** Pyrazines contents (µg/g) in the model system (glucose with lysine-containing tripeptides) and in the Control _(Arg + Lys+ Lys)_ (*p <* 0.05). L-A-H: Lys-Arg-His; L-H-A: Lys-His-Arg; H-A-L: His-Arg-Lys; H-L-A: His-Lys-Arg; A-L-H: Arg-Lys-His; A-H-L: Arg-His-Lys.

No.	RT (min)	Substances	L-A-H	L-H-A	H-A-L	H-L-A	A-L-H	A-H-L	A+H+L
1	9.556	pyrazine	0.05 ± 0.002	0.04 ± 0.002	0.02 ± 0.001	0.02 ± 0.001	0.03 ± 0.001	0.04 ± 0.003	0.12 ± 0.02
2	10.907	2-methylpyrazine	0.52 ± 0.02	0.43 ± 0.03	0.22 ± 0.03	0.22 ± 0.02	0.50 ± 0.03	0.52 ± 0.05	1.90 ± 0.12
3	12.649	2,5(6)-dimethylpyrazine	1.63 ± 0.04	1.41 ± 0.17	0.69 ± 0.18	0.61 ± 0.01	1.80 ± 0.13	1.83 ± 0.12	4.68 ± 0.11
4	13.057	2-ethylpyrazine	0.11 ± 0.01	0.09 ± 0.01	0.06 ± 0.002	0.04 ± 0.001	0.13 ± 0.004	0.14 ± 0.03	ND
5	13.516	2,3-dimethylpyrazine	0.24 ± 0.02	0.20 ± 0.02	0.02 ± 0.001	0.02 ± 0.001	0.06 ± 0.005	0.06 ± 0.003	0.08 ± 0.01
6	14.858	2-ethyl-6-methylpyrazine	0.13 ± 0.01	0.12 ± 0.01	0.10 ± 0.007	0.14 ± 0.02	0.40 ± 0.002	0.24 ± 0.002	1.15 ± 0.08
7	15.092	2-ethyl-5-methylpyrazine	0.85 ± 0.02	0.77 ± 0.05	0.32 ± 0.02	0.29 ± 0.06	0.91 ± 0.09	0.95 ± 0.01	1.02 ± 0.03
8	15.525	2,3,5-trimethylpyrazine	0.43 ± 0.01	0.40 ± 0.03	0.08 ± 0.003	0.09 ± 0.001	0.32 ± 0.08	0.32 ± 0.02	0.77 ± 0.01
9	15.975	2-(*N*-propyl)pyrazine	ND	ND	ND	ND	0.01 ± 0.001	ND	ND
10	16.484	2,6-diethylpyrazine	1.99 ± 0.04	0.02 ± 0.001	0.01 ± 0.001	0.01 ± 0.001	0.04 ± 0.002	0.03 ± 0.001	0.03 ± 0.002
11	16.534	ethenylpyrazine	ND	ND	ND	ND	ND	ND	0.05 ± 0.001
12	16.834	3-ethyl-2,5-dimethylpyrazine	0.31 ± 0.01	0.25 ± 0.02	0.11 ± 0.01	0.16 ± 0.03	0.48 ± 0.05	0.31 ± 0.005	1.02 ± 0.01
13	17.334	2,5-diethylpyrazine	0.13 ± 0.03	0.10 ± 0.01	0.04 ± 0.003	0.05 ± 0.003	0.12 ± 0.01	0.12 ± 0.002	0.16 ± 0.01
14	17.376	2-ethyl-3,5-dimethylpyrazine	0.21 ± 0.07	0.19 ± 0.04	0.03 ± 0.001	0.03 ± 0.002	0.15 ± 0.02	0.12 ± 0.001	ND
15	17.476	2-methyl-6-propylpyrazine	0.01 ± 0.001	ND	ND	0.01 ± 0.001	0.02 ± 0.001	ND	0.12 ± 0.008
16	17.859	2-methyl-5-propylpyrazine	0.02 ± 0.001	0.03 ± 0.002	ND	ND	0.03 ± 0.001	0.02 ± 0.001	ND
17	18.201	2-ethenyl-6-methylpyrazine	ND	ND	ND	ND	0.01 ± 0.001	0.01 ± 0.001	0.08 ± 0.002
18	18.359	3,5-diethyl-2-methylpyrazine	0.17 ± 0.03	0.13 ± 0.01	ND	0.08 ± 0.005	ND	0.14 ± 0.002	0.16 ± 0.01
19	18.960	2,5-dimethyl-3-propylpyrazine	0.04 ± 0.002	0.02 ± 0.01	ND	ND	ND	0.02 ± 0.001	0.16 ± 0.02
20	19.027	2,3-diethyl-5-methylpyrazine	0.02 ± 0.001	ND	ND	ND	ND	ND	ND
21	19.685	2-methyl-6-(1-propenyl)-, (E)-pyrazine	ND	ND	ND	ND	ND	0.02 ± 0.001	0.29 ± 0.01
		Total pyrazines	6.86 ± 0.11	4.20 ± 0.21	1.70 ± 0.06	1.77 ± 0.004	5.01 ± 0.15	4.89 ± 0.32	11.79 ± 0.98
		Pyrazines (%of total GC–MS peak area)	79.16	72.78	13.84	27.90	81.30	69.48	30.38

ND represents no substance was detected.
